# Efficient Visible Light Communication Transmitters Based on Switching-Mode dc-dc Converters

**DOI:** 10.3390/s18041127

**Published:** 2018-04-07

**Authors:** Juan Rodríguez, Diego G. Lamar, Daniel G. Aller, Pablo F. Miaja, Javier Sebastián

**Affiliations:** 1 Power Supply Group, Electrical Engineering Department, University of Oviedo, Gijón 33204, Spain; gonzalezdiego@uniovi.es (D.G.L.); garciaadaniel@uniovi.es (D.G.A.); sebas@uniovi.es (J.S.); 2 Power Systems Division, European Space Agency (ESA), Noordwijk 2201AZ, The Netherlands; Pablo.Fernandez@esa.int

**Keywords:** visible light communication (VLC), light emitting diode (LED), switching-mode dc-to-dc converter (SMC_dc-dc_)

## Abstract

Visible light communication (VLC) based on solid-state lighting (SSL) is a promising option either to supplement or to substitute existing radio frequency (RF) wireless communication in indoor environments. VLC systems take advantage of the fast modulation of the visible light that light emitting diodes (LEDs) enable. The switching-mode dc-to-dc converter (SMC_dc-dc_) must be the cornerstone of the LED driver of VLC transmitters in order to incorporate the communication functionality into LED lighting, keeping high power efficiency. However, the new requirements related to the communication, especially the high bandwidth that the LED driver must achieve, converts the design of the SMC_dc-dc_ into a very challenging task. In this work, three different methods for achieving such a high bandwidth with an SMC_dc-dc_ are presented: increasing the order of the SMC_dc-dc_ output filter, increasing the number of voltage inputs, and increasing the number of phases. These three strategies are combinable and the optimum design depends on the particular VLC application, which determines the requirements of the VLC transmitter. As an example, an experimental VLC transmitter based on a two-phase buck converter with a fourth-order output filter will demonstrate that a bandwidth of several hundred kilohertz (kHz) can be achieved with output power levels close to 10 W and power efficiencies between 85% and 90%. In conclusion, the design strategy presented allows us to incorporate VLC into SSL, achieving high bit rates without damaging the power efficiency of LED lighting.

## 1. Introduction

Visible light communication (VLC) is a particular case of optical wireless communication (OWC) that uses the intensity of visible light beams to transmit information [1,2,3]. Recent advances in light emitting diodes (LEDs) have pushed up the interest in combining VLC and solid-state lighting (SSL) either to supplement or to substitute existing radio frequency (RF) wireless communication systems for indoor environments because of its main benefits:

- VLC opens up a large license-free visible region for wireless communication in comparison to the restricted and expensive RF spectrum.
- No special facilities are needed to implement the VLC systems because, in principle, they can be integrated into the existing lighting ones.
- Since visible light cannot penetrate building walls, VLC provides communication security and naturally avoids interference with other VLC systems from adjacent rooms, thus providing the entire available bandwidth for each closed environment.

Obviously, VLC has some drawbacks that should be pointed out:

- Although line-of-sight is not mandatory for enabling VLC (reflected beams keep transmitting the information), the performance falls and it is strongly jeopardized by the presence of obstacles.
- The range of VLC systems is lower than that of RF systems.

Taking into account the aforementioned characteristics, the use of VLC has been proposed in several applications:

- Internet connectivity in indoor scenarios where the existing light infrastructure can be adapted [4,5].
- Vehicle to vehicle communications where VLC can be implemented using car headlights [6,7].
- Underwater communications where light is less attenuated than RF signals [8,9].
- Wireless communications in scenarios where RF communication is dangerous, such as airplanes, mines, and hospitals [10].

LEDs generate light based on an electroluminescence phenomenon that occurs when an electric field is applied to a P–N junction, promoting the recombination of electrons and holes. Since LEDs are made with direct semiconductor materials, the recombination generates photons in a determined wavelength and heat. The LED implementation for lighting applications is normally executed with a semiconductor GaN die, which emits blue light that excites a phosphor. A part of the blue photons travels through the phosphor layer without alteration, whereas the rest are converted into yellow photons. Therefore, the white light that is finally emitted by the LED is a combination of blue and yellow photons. Due to their nature, LEDs are power efficient, environmentally friendly, and reliable. For these reasons, LEDs have become strong in the lighting market compared to traditional lighting solutions. In addition, they provide an essential characteristic to enable VLC implementation: the intensity of the light emitted by a LED can be modulated significantly quickly.

If a lighting LED is used to transmit information, the fast changes of the light intensity demanded by the communication functionality (*s_LED−ac_*(*t*)) must be added to the constant light intensity value (*s_LED−dc_*) that is characteristic of the lighting function:

(1) $$s_{LED}\left(t\right)=s_{LED-dc}+s_{LED-ac}\left(t\right).$$

Considering the nature of the LED, the light intensity modulation over a certain constant light intensity is translated into a current modulation over a certain constant current. Therefore, the current through the LEDs must be made up of a dc component (*i_LED−dc_*) that performs the lighting function, plus an ac component (*i_LED−ac_*(*t*)) that represents the modulated information signal.

The information can be transmitted using two different modulation strategies: pulse modulation (PM) and pass-band modulation (PBM).

**Figure 1:**
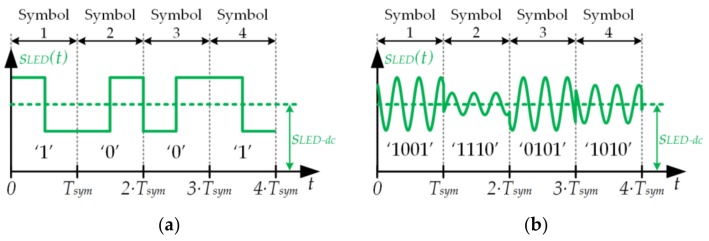
Two modulating strategies for visible light communication (VLC): (**a**) Example of a pulse modulation sequence: four symbols of a pulse position modulation (PPM) scheme; (**b**) Example of a pass-band modulation sequence: four symbols of a 16-quadrature amplitude modulation (16-QAM).

- In the case of PM, *s_LED−ac_*(*t*) is a pulse waveform that is added to the lighting level (i.e., *s_LED−dc_*). On-off keying (OOK) and pulse position modulation (PPM) are examples of PM schemes that are commonly used in VLC. Figure 1a shows an example of a PPM sequence that is made up of four symbols. Note that the position of the pulse at the beginning or at the end of the symbol period (*T_Sym_*) determines the transmitted bit. Strategies based on PM are very simple to implement, but they are inefficient from the communication perspective because they have two problems. The first one is that for a given bandwidth they cannot achieve bit rates as high as the PBM schemes that are explained below. The second problem is that they are hardly jeopardized by the different trajectories that the visible light beams follow before reaching the receiver (i.e., multipath issues).
- In the case of PBM, *s_LED−ac_*(*t*) is a sinusoid or the sum of several sinusoids that change the amplitude and/or the phase over time according to the information that is being transmitted. This strategy achieves higher bit rates than PM for a given bandwidth and, as will be detailed below, there are certain PBM schemes that provide high immunity against the multipath issue. If the PBM scheme is made up of a single modulated sinusoid, the modulation scheme is referred to as a single-carrier modulation (SCM) scheme. The three main SCM schemes are amplitude-shift keying (ASK), where the amplitude changes over time; phase-shift keying (PSK), where the phase changes over time; and quadrature amplitude modulation (QAM), where both the amplitude and the phase change over time. Figure 1b shows an example of a 16-QAM sequence.

As previously indicated, the pass-band signal can be made up of several sinusoids that change the amplitude and/or the phase over time. In this case, the strategy is referred to as a multi-carrier modulation (MCM). Discrete multitone modulation (DMT) and orthogonal frequency division multiplexing (OFDM) are two MCM schemes that are commonly used in VLC systems. MCM schemes are the preferred modulation strategies for VLC because they are robust against multipath issues [11,12,13].

Regardless of the number of carriers, any PBM signal can be expressed as a single modulated sinusoid:

(2) $$s_{LED-AC}\left(t\right)=A_{s}\left(t\right)\cdot \cos\left(2\cdot \pi \cdot f_{o}\cdot t+\phi_{s}\left(t\right)\right),$$

where *f_o_* is the frequency of the sinusoid (i.e., the center frequency of the pass-band signal), *A_s_*(*t*) is the modulated amplitude, and *ϕ_s_*(*t*) is the modulated phase.

**Figure 2:**
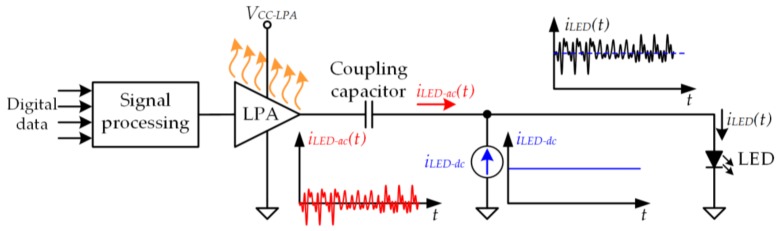
Conventional architecture of a VLC transmitter.

Figure 2 shows the conventional architecture of a VLC transmitter [3,5,14,15,16,17,18,19,20,21,22,23]. The LED driver is based on the use of a linear power amplifier (LPA) operating in class A, class B, or class AB that delivers *i_LED−ac_*(*t*). Moreover, a dc current source injects *i_LED−dc_* to adequately bias the LED. Since the LPA is only able to deliver a low power level, the architecture has a single LED and, consequently, the range of the VLC system is strongly reduced (units of centimeter). In addition, the power efficiency of an LPA ranges between 10% and 40% depending on the operating class and the modulation scheme that is being reproduced. Therefore, it consumes a lot of power and, as a result, the use of large and expensive cooling systems (i.e., heat sink, fan, etc.) to extract the heat from the semiconductor devices is mandatory. However, the LPA has two characteristics that explain why it is widely used for driving the LEDs for VLC: it achieves a very high bandwidth and it has a linear relationship between its input and its output. Both virtues ensure an accurate reproduction of the communication signal.

In the case of short-range applications that demand low power levels, low power efficiency can be affordable. However, if the power of the communication signal is increased (with the purpose of increasing the link distance: tens of centimeters to several meters), a circuit able to drive the LEDs achieving high efficiency is essential. Using a power-inefficient LED driver damages one of the reasons why LED lighting is replacing incandescent lighting: the high power efficiency. A key point that is generally not addressed in VLC literature is that the high efficiency of LED lighting is not only due to the high efficiency of LEDs converting electrical power into optical power, but also due to the high efficiency achieved by the LED driver that delivers the electrical power.

In conclusion, a power-efficient LED driver for VLC transmitters is mandatory for increasing the power of the communication signal in order to enable a higher communication range [24]. Switching-mode converters (SMCs) are the cornerstones of the LED drivers used in conventional lighting applications. They reach a very high efficiency (>90%) [25] and, therefore, they are a very interesting option to be used in LED drivers for VLC. However, major challenges must be addressed, such as the high bandwidth demanded by the LED driver to fulfill the VLC requirements.

## 2. Fundamentals of Driving the LEDs of VLC with Switching-Mode dc-dc Converters

### 2.1. The Role of Switching-Mode Converters in Solid-State Lighting

**Figure 3:**
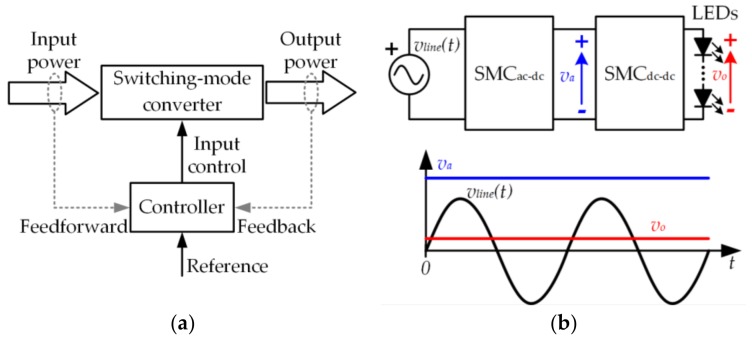
(**a**) Basic power processing block of a switching-mode converter; (**b**) Two-stage power conversion architecture of solid-state lighting (SSL).

SMCs change the format of the power, which is univocally defined by a combination of voltage and current. In general, an SMC is made up of three ports: an input power port, a control port, and an output power port (see Figure 3a). The great benefit of SMCs is that they are able to perform the conversion of the power format (from the input to the output) dissipating very little power. Therefore, the power efficiency achieved by an SMC is very high (between 85% and 95% depending on the application). Note that the power efficiency (*η*) is defined as the ratio between the output power (*P_o_*) and the input power (*P_in_*):

(3) $$\eta =\frac{P_{o}}{P_{in}}.$$

The use of non-dissipative elements in the SMC (i.e., capacitors, inductors, and switching-mode semiconductor devices) is the cornerstone for achieving high power efficiency. Four sub-categories of SMCs can be defined according to the kind of power conversion performed:

- The switching-mode dc-to-dc converter (SMC_dc-dc_) converts a direct input voltage into a different direct output voltage.
- The switching-mode dc-to-ac converter (SMC_dc-ac_) converts a direct input voltage into an alternating output voltage.
- The switching-mode ac-to-dc converter (SMC_ac-dc_) converts an alternating input voltage into a direct output voltage.
- The switching-mode ac-to-ac converter (SMC_ac-ac_) converts an alternating input voltage into a different alternating output voltage.

Figure 3b shows one of the most extended power conversion architectures used in SSL. In this case, the conversion of the power format is performed in two stages. In the first stage, the ac line voltage (*v_line_*)(*t*) is converted into a constant voltage (*v_a_*). Since *v_a_* is too high for directly driving the LEDs, a second stage is used to convert *v_a_* into the required constant voltage (*v_o_*).

### 2.2. Incorporating VLC into SSL

**Figure 4:**
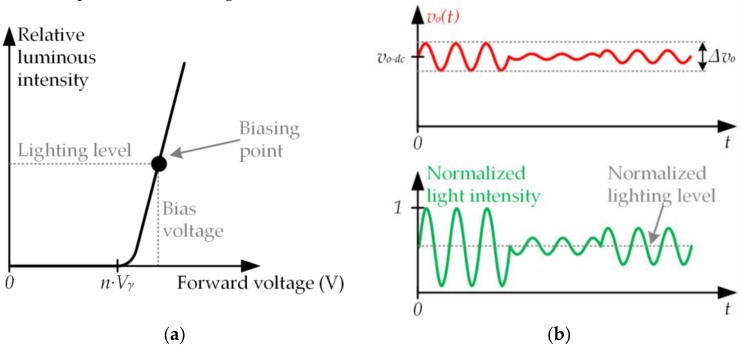
(**a**) Relationship between the light intensity emitted by a string of *n* LEDs and the applied voltage; (**b**) Example of the involved voltage and normalized light intensity waveforms when a pass-band signal is reproduced.

To properly modify the existing SSL infrastructure to incorporate VLC, the new requirements of the LED driver must be taken into account. Figure 4a shows the relationship between the light intensity emitted by a string of *n* LEDs and the applied voltage (i.e., *v_o_*(*t*)). It can be seen that the LED string starts to emit light once *v_o_*(*t*) overcomes the following voltage threshold:

(4) $$V_{th}=n\cdot V_{\gamma },$$

where *V_γ_* is the knee voltage of each LED. Moreover, there is a linear relationship between light intensity and voltage when *v_o_*(*t*) > *n*·*V_γ_*. This characteristic is very important because it implies that the information transmission can be performed by generating voltage variations proportional to the desired light intensity variations. Therefore, in the case of incorporating VLC into SSL, the voltage applied to a LED string must be made up of a dc component (*v_o−dc_*) that determines the lighting level and an ac component (*v_o−ac_*(*t*)) that is related to the communication signal:

(5) $$v_{o}\left(t\right)=v_{o-dc}+v_{o-ac}\left(t\right)=v_{o-dc}+A_{v}\left(t\right)\cdot \cos\left(2\cdot \pi \cdot f_{o}+\phi_{v}\left(t\right)\right),$$

where *A_v_*(*t*) is the modulated amplitude and *ϕ_v_*(*t*) is the modulated phase. According to (2) and to the linear relationship between the light intensity and the applied voltage, *A_v_*(*t*) is proportional to *A_s_*(*t*) while *ϕ_v_*(*t*) is equal to *ϕ_s_*(*t*) (see Figure 4b).

**Figure 5:**
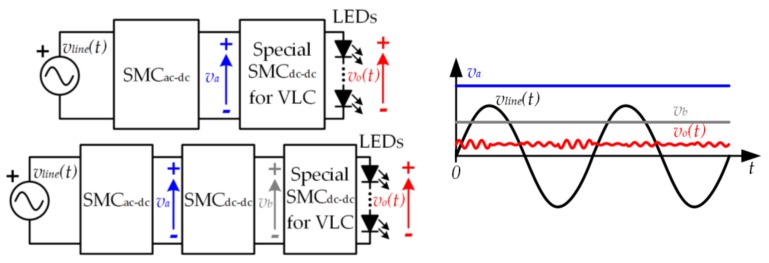
Modification of the existing SSL infrastructure to incorporate VLC while keeping high power efficiency.

Although the SMC_dc-dc_ is used mostly to provide a constant output voltage, it is also able to generate a variable output voltage (i.e., it is able to reproduce (5)). Therefore, a power-efficient SMC_dc-dc_ can be used for completely driving the LED string (i.e., lighting and communication), thus avoiding the use of any inefficient LPA. However, this SMC_dc-dc_ is very different from conventional SMCs used in lighting applications, such as the one depicted in Figure 3b, and, as will be detailed in the next section, the design becomes very challenging [26,27,28]. In any case, there are two main options for modifying the SSL architecture to incorporate VLC while keeping the use of SMCs. The first one consists in replacing the conventional SMC_dc-dc_ used at the last stage of the SSL architecture by the SMC_dc-dc_ specially designed for VLC. Therefore, this SMC_dc-dc_ must provide the bias level (i.e., *v_o−dc_*) and the small voltage variations related to the communication signal (i.e., *v_o−ac_*(*t*)) from a much higher voltage (i.e., *v_a_*). However, generating very small voltage variations from a much higher voltage is quite difficult using an SMC_dc-dc_. This problem can be alleviated by modifying the SSL architecture as follows. As in the existing SSL infrastructure, an SMC_ac-dc_ converts *v_line_*(*t*) into *v_a_*. Then, a conventional SMC_dc-dc_ (similar to the one depicted in Figure 3b) converts *v_a_* into a lower constant voltage (*v_b_*) that is closer to the desired *v_o_*(*t*) and always higher than that one (i.e., always satisfying *v_b_* > *v_o_*(*t*)). Finally, an SMC_dc-dc_ specially designed for VLC provides *v_o_*(*t*) from *v_b_* (see Figure 5).

### 2.3. Buck Converter: The Backbone of the SMC_dc-dc_ Specially Designed for VLC

**Figure 6:**
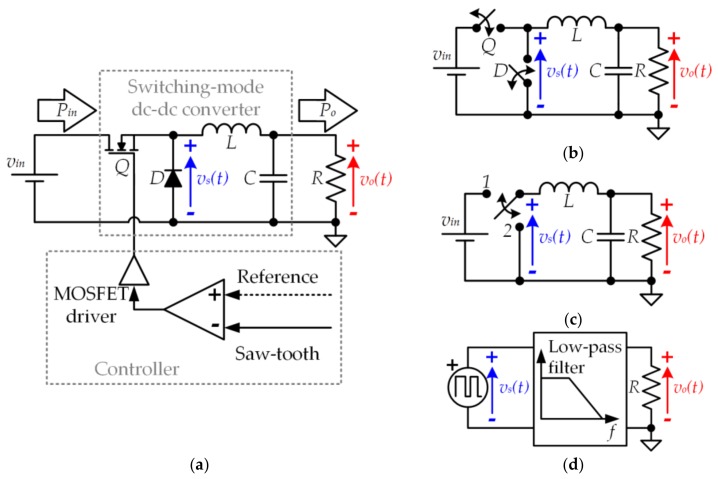
(**a**) Buck converter; (**b**) Equivalent circuit considering ideal complementary switches; (**c**) Equivalent circuit considering the two possible operating states; (**d**) The buck converter seen as a pulse-width modulator.

The buck converter is the fundamental SMC_dc-dc_ and, as will be explained in the following sections, it is the cornerstone of the special SMC_dc-dc_ designs for VLC that are proposed in this paper. The buck converter transforms the input voltage (*v_in_*) into a lower one (i.e., *v_o_*(*t*) < *v_in_*). Figure 6 shows the conventional buck converter topology and the equivalent circuits that will support the explanation of its operating principle.

When the metal-oxide-semiconductor field-effect transistor (MOSFET) is activated (state 1), it behaves as a short circuit and, consequently, the voltage applied to the input of the L-C filter (*v_s_*)(*t*) is equal to *v_in_*. Note that in this state, the diode is deactivated and blocks *v_in_*. Moreover, when the MOSFET is deactivated (state 2), the diode conducts the inductor current, operating as a short circuit. Therefore, *v_s_*(*t*) is equal to 0 V during this state. In conclusion, the MOSFET and the diode operate as complementary switches (see Figure 6b) and the buck converter has two operating states (see Figure 6c). Since the buck converter continually alternates between state 1 and state 2, it can be seen as a pulse voltage waveform with amplitude *v_in_* that is applied to a low-pass filter. Essentially, the operating principle of the buck converter is based on the pulse-width modulation (PWM) technique (see Figure 6d). In this way, the ratio between the time that the buck converter remains in state 1 and the switching period (*T_s_*) determines the output voltage:

(6) $$v_{o}\left(t\right)=d\left(t\right)\cdot v_{in},$$

where *d*(*t*) is the duty cycle, which is the term commonly used to denote the aforementioned ratio.

**Figure 7:**
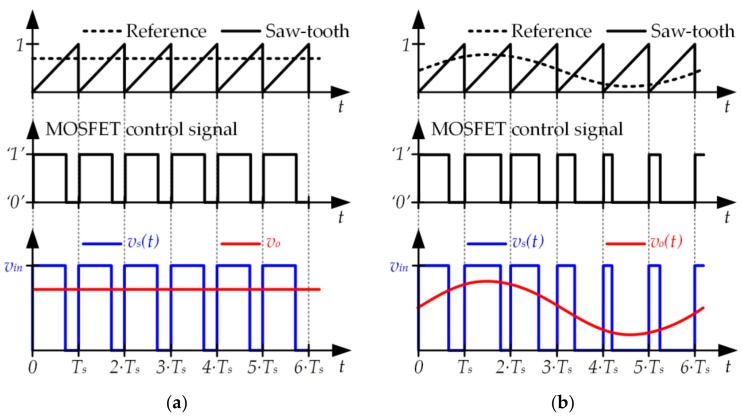
(**a**) Buck converter tracking a constant reference. (**b**) Buck converter tracking a variable reference.

As previously indicated, the typical target of the buck converter is to provide a constant output voltage (see Figure 7a) by tracking a constant reference. In this case, the duty cycle (i.e., the width of the pulses) remains constant over time. However, there are special applications of power electronics where a buck converter is used to track a variable reference (see Figure 7b). In this case, the duty cycle changes over time according to the reference that is being tracked. Therefore, the buck converter operates as a power amplifier where the reference, *v_o_*(*t*), and *v_in_* are the input, the output, and the voltage gain, respectively.

### 2.4. Design Challenges of Using a Buck Converter as the LED Driver of a VLC Transmitter

In practice, any SMC is made up of real components that include parasitic elements. Hence, SMCs have several sources of power loss and, as a result, the 100% theoretical efficiency is never reached. In the case of using a buck converter to drive the LEDs of a VLC transmitter, the switching losses of the switching-mode semiconductor devices are the most critical problem. The transition of a real switching-mode semiconductor device (either a MOSFET or a diode) between on-state (i.e., the device is activated) and off-state (i.e., the device is deactivated) is not instantaneous. During transitions, the switching-mode semiconductor device dissipates power since it is blocking voltage and driving current at the same time. Obviously, switching losses rise with the switching frequency (*f_s_*) of the buck converter, which can jeopardize the power efficiency. This is not a problem in the case of the conventional SMC_dc-dc_ used is SSLs where *f_s_* ranges between the tens and the hundreds of kilohertz. However, switching losses become critical when *f_s_* reaches the megahertz (MHz) range and, as will be explained below, this is the situation that occurs when a buck converter is used to drive the LED string of a VLC transmitter.

According to the previous sections, the voltage waveform indicated in (5) can be provided by a buck converter. However, since the maximum frequency of *v_o_*(*t*) (*f_o−max_*) could be relatively high (reaching the megahertz range when the target is to provide a very high bit rate), a very fast LED driver is mandatory. Since the buck converter acts as a pulse-width modulator, the Nyquist–Shannon sampling theorem establishes the theoretical minimum switching frequency that can be used:

(7) $$f_{s-min}=2\cdot f_{o-max}.$$

However, implementing a buck converter with *f_s_* equal to *f_s−min_* is impractical because it would cause too much distortion of the communication signal due to the output voltage ripple. In practice, *f_s_* must be around 20·*f_o−max_* to achieve enough rejection of the switching harmonics [29]. In the case of using a buck converter as the LED driver of a VLC transmitter, the aforementioned ratio between *f_o−max_* and *f_s_* is translated into unaffordable switching losses that jeopardize the implementation. In fact, the problem is not only the low power efficiency that would be achieved (probably below 80%), but also the malfunction that the power dissipation would cause to the switching-mode semiconductor devices.

**Figure 8:**
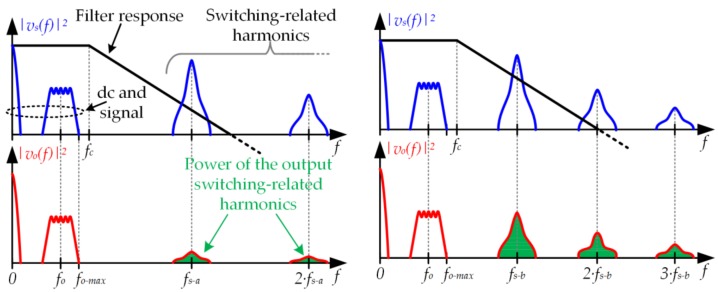
Analysis in the frequency domain of the distortion increase when *f_s_* is reduced (*f_s-b_* < *f_s-a_*).

Figure 8 exemplifies the problem considering two switching frequency values (*f_s-a_* > *f_s-b_*) in order to highlight the impact of reducing *f_s_*. If *f_s_* is reduced (i.e., from *f_s-a_* to *f_s-b_*), switching losses fall and, as a result, the efficiency of the buck converter rises. However, since the switching-related harmonics are closer to the cut-off frequency of the filter (*f_c_*), they are rejected in a minor manner and, consequently, the distortion of the signal at the output will be higher. Note that in order to maximize the rejection of the switching harmonics, *f_c_* must be the lowest value that allows the pass of the communication signal without distorting it (regardless of the selected *f_s_*).

In conclusion, although the buck converter can theoretically be used as the LED driver of a VLC transmitter, in practice, the required *f_s_* makes the approach impractical.

## 3. Buck-Derived dc-dc Converters, a Clever Solution to Drive the LEDs of VLC Transmitters

To reduce the gap between *f_o−max_* and *f_s_* without increasing the distortion caused by the switching-related harmonics, more sophisticated solutions than the conventional buck converter are mandatory. Several topologies derived from the buck converter that have been proposed for other applications of power electronics are reviewed in this section. The design modifications add certain features that make the topologies very interesting for the LED driver of VLC transmitters. In addition, these three strategies are combinable [30,31] and the optimum design depends on the particular VLC application, which determines the requirements of the VLC transmitter.

### 3.1. Buck Converter with a High-Order Output Filter

**Figure 9:**
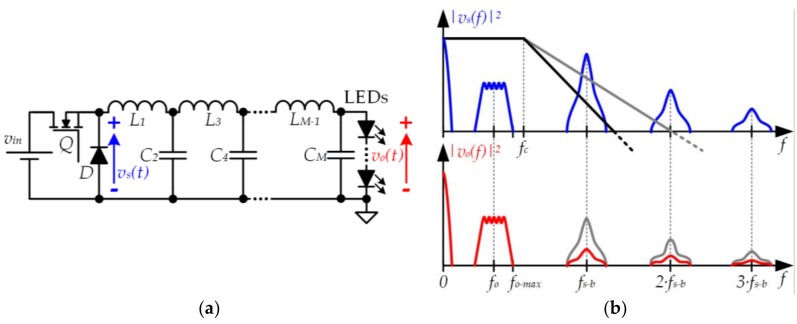
(**a**) Buck converter with *M^th^*-order output filter; (**b**) The output voltage ripple can be reduced by increasing the filter order.

A straightforward method to reduce the gap between *f_o−max_* and *f_s_* without increasing the distortion is to increase the order of the buck converter filter (see Figure 9a) [32,33,34,35,36,37,38,39,40]. For a certain *f_s_* value, the higher the filter order, the higher the rejection of the switching-related harmonics (see Figure 9b).

### 3.2. Two-Input Buck Converter

**Figure 10:**
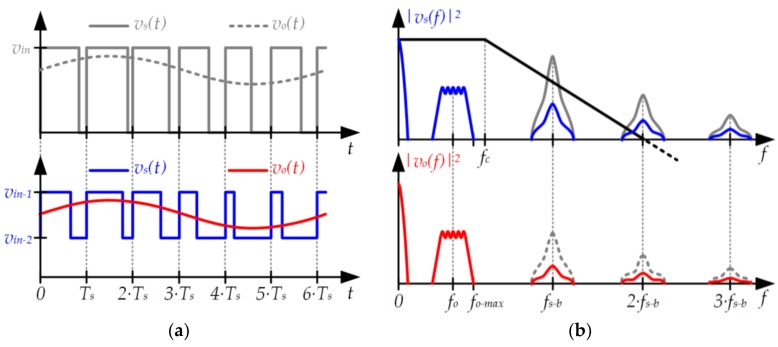
(**a**) Pulse-width modulator using a pulse voltage waveform that alternates between two positive voltage values (*v*_*in*−1_ and *v*_*in*−2_); (**b**) Reduction of the switching-related harmonics at the filter input and, as a consequence, at the filter output.

The second approach is focused on reducing the power of the switching-related harmonics at the input of the filter. Since the input of the buck filter is a PWM voltage waveform (i.e., *v_s_*)(*t*), the switching-related harmonics at the input of the filter are determined by the amplitude of the pulses. The lower the amplitude of the pulses, the lower the power of the switching-related harmonics at the input of the filter. To properly address this point, the variation range of *v_o_*(*t*) should be characterized. According to Section 2.1 and as Figure 4 shows, the variable component of *v_o_*(*t*) (i.e., *v_o−ac_*(*t*)) is relatively small in comparison to the continuous component (i.e., *v_o−dc_*). Therefore, the pulse amplitude can be reduced by using a positive value instead of 0 V when the pulse voltage waveform is in a low state. In this case, the output voltage follows this expression:

(8) $$v_{o}\left(t\right)=d\left(t\right)\cdot \left(v_{in-1}-v_{in-2}\right)+v_{in-2},$$

where *v_in−1_* and *v_in−2_* are the highest and lowest value of the pulse voltage waveform, respectively. Figure 10 exemplifies this approach and shows the reduction of the switching-related harmonics achieved at the filter input and, as a result, at the filter output.

**Figure 11:**
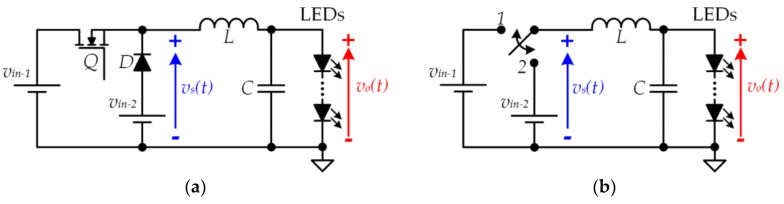
(**a**) Two-input buck converter; (**b**) Equivalent circuit considering the two possible operating states.

The modification of the conventional buck converter required to generate the pulse voltage waveform that alternates between *v*_*in*−1_ and *v*_*in*−2_ was introduced in [41]. Later, this new SMC_dc-dc_ topology, which is known as a two-input buck converter (see Figure 11), was extended to form a multiple-input buck converter [42,43].

### 3.3. Two-Phase Buck Converter

**Figure 12:**
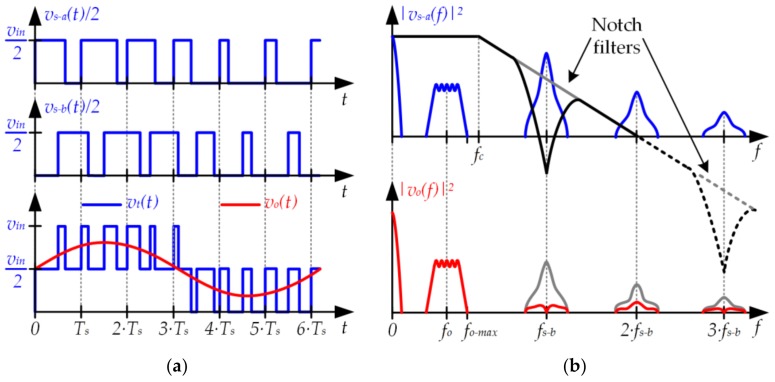
(**a**) Pulse-width modulator using two identical pulse voltage waveforms but with 180° out of phase; (**b**) Reduction of the switching-related harmonics.

The third approach is also focused on reducing the power of the switching-related harmonics at the input of the filter. In this case, the strategy is to sum two pulse voltage waveforms (*v_s−a_*(*t*) and *v_s−b_*(*t*)) in order to obtain a third one (*v_t_*(*t*)) that has less power of the switching-related harmonics. In particular, and as Figure 12a shows, the method consists in making *v_s−b_*(*t*) equal to *v_s−a_*(*t*) with 180° out of phase (i.e., there is a delay of *Ts*/2 between *v_s−a_*(*t*) and *v_s−b_*(*t*)) and dividing the result by two. In this way, the harmonic content around the odd switching harmonics (i.e., around *f_s_*, *3·f_s_*, *5·f_s_*, etc.) is much lower than in the case of the conventional buck converter.

The pulse voltage waveforms must be mathematically modeled in order to deeply understand the improvement achieved. *v_t_*(*t*) can be expressed as follows:

(9) $$v_{t}\left(t\right)=\frac{1}{2}\cdot \left[v_{s-a}\left(t\right)+v_{s-b}\left(t\right)\right].$$

Taking into account that *v_s−b_*(*t*) can be written as a function of *v_s−a_*(*t*), (9) can be rewritten as follows:

(10) $$v_{t}\left(t\right)=\frac{1}{2}\cdot \left[v_{s-a}\left(t\right)+v_{s-a}\left(t-\frac{T_{s}}{2}\right)\right].$$

In the frequency domain, (10) can be expressed as:

(11) $$v_{t}\left(f\right)=v_{s-a}\left(f\right)\cdot \frac{\left(1+e^{-j\cdot \pi \cdot T_{s}\cdot f}\right)}{2}=v_{s-a}\left(f\right)\cdot H\left(f\right),$$

where *H*(*f*) is the transfer function between *v_s−a_*(*t*) and *v_t_*(*t*):

(12) $$H\left(f\right)=\frac{\left(1+e^{-j\cdot \pi \cdot T_{s}\cdot f}\right)}{2}.$$

**Figure 13:**
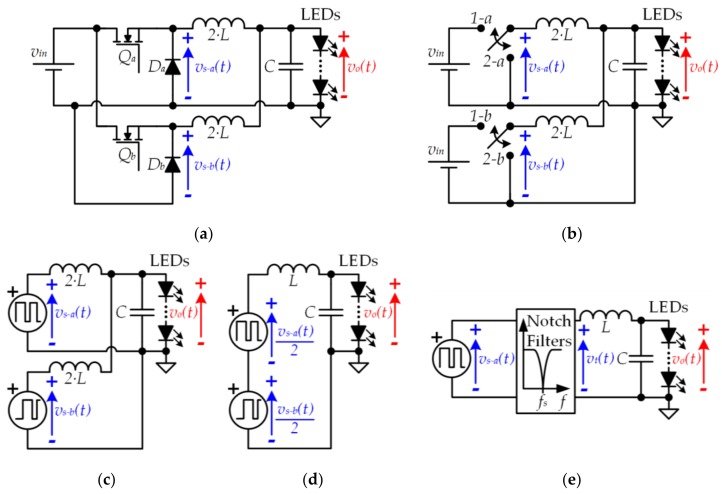
(**a**) Two-phase buck converter; (**b**) Equivalent circuit considering the two possible operating states; (**c**) Equivalent circuit considering two pulse-width modulators; (**d**) Equivalent circuit obtained by applying the superposition and Thevenin’s theorem to the capacitor terminals; (**e**) Equivalent circuit considering *H*(*f*).

As Figure 12b shows, *H*(*f*) introduces notch filters centered at odd switching harmonics, thus reducing their power. The aforementioned approach can be implemented using a two-phase buck converter (see Figure 13), which is a particularization of the multi-phase buck converter [32,33,34,35,44,45,46,47,48,49,50].

## 4. Experimental Section

**Figure 14:**
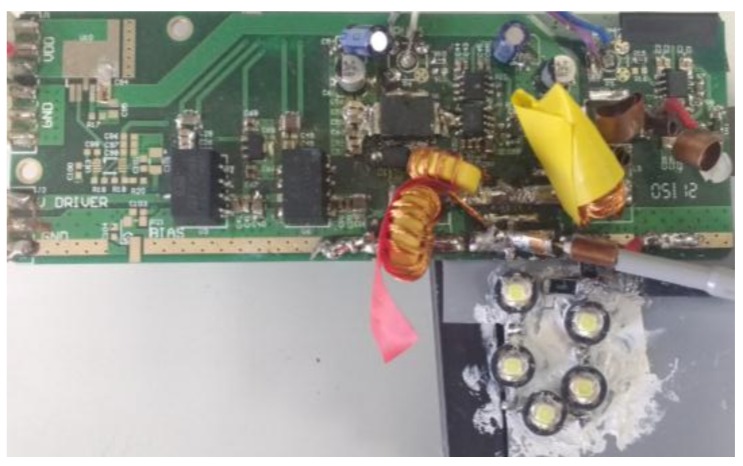
Prototype of the implemented two-phase buck converter with a fourth-order output filter and five LEDs.

Figure 14 shows the prototype of a VLC transmitter that was designed following two of the aforementioned strategies. In particular, the LED driver is a two-phase buck converter with a fourth-order output filter. The input voltage is 33 V and the load is made up of five LEDs (W42180 from Seoul Semiconductor (Ansan, South Korea)).

In order to bias the LED string in the linear region, *v_o−dc_* equal to 16.5 V was chosen. The average current through the load is 0.5 A, which determines the lighting level. The current through the load is measured using a precision shunt resistor of 0.75 Ω.

**Figure 15:**
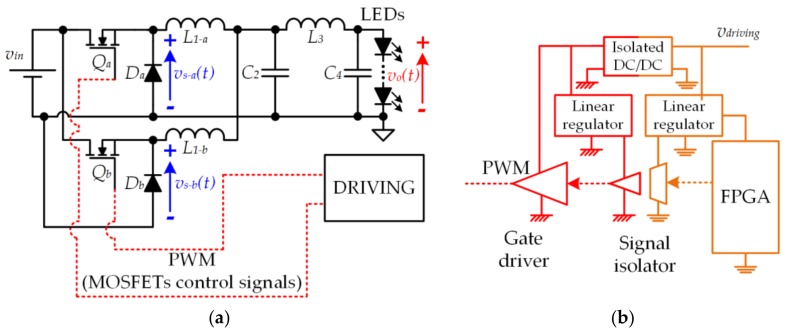
(**a**) Circuit of the two-phase buck converter with a fourth-order output filter; (**b**) Driving system and auxiliary power supply of each phase.

Figure 15a,b shows the power stage of the converter and the driving circuit of each phase, respectively. Due to the high switching frequency value (*f_s_* = 4.5 MHz), the choice of the components is critical. A RF transistor PD55008 Si-LDMOS (i.e., Q_a_ and Q_b_) from STMicroelectronics (Geneva, Switzerland) and a diode MBRS14 (i.e., D_a_ and D_b_) from ON Semiconductor (Phoenix, AZ, USA) were chosen for the power stage. An isolated driving stage is necessary for this topology due to the floating position of the MOSFETs. The driving circuit is made up of the gate driver EL7155 from Intersil (Milpitas, CA, USA) and the digital isolator ISO7220 from Texas Instruments (Dallas, TX, USA). The floating power supply is implemented using an isolated dc-dc converter ISF1209 A from XP Power (Singapore). Finally, The PWM signals used to drive the converter are calculated by MATLAB and generated by a Basys 2 FPGA from Digilent (Pullman, WA, USA).

### 4.1. Modulation Scheme

To evaluate the communication capability of the two-phase buck converter, a 16-QAM scheme with *f_o_* = 500 kHz was reproduced. Each symbol transmits four bits during four signal periods (i.e., *T_Sym_* = 8 μs), providing a bit rate equal to 500 kbps. Table 1 shows the normalized amplitude, phase, and bit code of each symbol. 

### 4.2. Filter Design

According to the previous design considerations, the cut-off frequency of the filter (i.e., *f_c_*) must be placed between the maximum frequency of the communication signal (i.e., *f_o−max_*) and the switching frequency (i.e., *f_s_*). The implemented output filter is a Legendre–Papoulis fourth-order filter with *f_c_* equal to 750 kHz. Table 2 shows the filter components.

### 4.3. Transmission Demonstration

**Figure 16:**
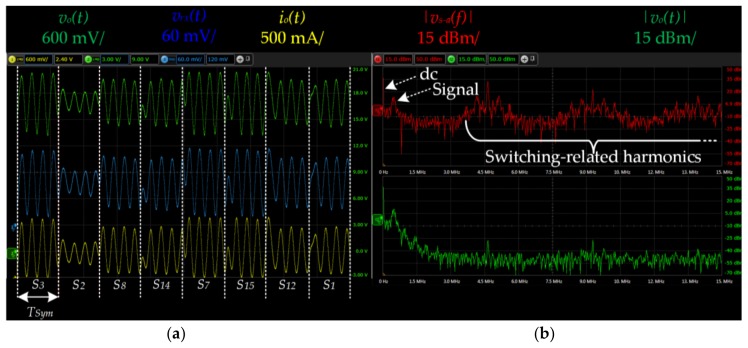
(**a**) Main waveforms of the transmitter where *v_o_*(*t*) is the voltage across the LEDs, *i_o_*(*t*) is the current through the LEDs and *v_rx_*(*t*) is the output voltage of the receiver. The link distance is 15 cm in order to show clear waveforms where the symbols can be easily identified; (**b**) Spectrum of *v_s−a_*(*t*) and *v_o_*(*t*).

Figure 16a shows an example of an eight-symbol sequence of the 16-QAM on the transmitter and receiver side. *v_o_*(*t*) shows the voltage across the LEDs, which has an average value equal to 16.5 V and a peak-to-peak value equal to 5 V. *i_o_*(*t*) shows the current through the LEDs, which has an average value of 0.5 mA and a peak-to-peak value equal to 0.9 A. *v_rx_*(*t*) shows the output voltage at the receiver. In order to measure the light signal of the LEDs, an optical receiver PDA10A-EC from Thorlabs (Newton, MA, USA) has been used. It is important to highlight that the three signals are proportional and there is no noticeable distortion because the LEDs are working in their linear region.

Figure 16b shows the spectrum of both *v_s−a_*(*t*) and *v_o_*(*t*). It is important to note that the dc component and the 16-QAM signal (around 500 kHz) pass through the filter without alteration, whereas the switching-related harmonics are attenuated due to the filter and the two-phase effect.

**Figure 17:**
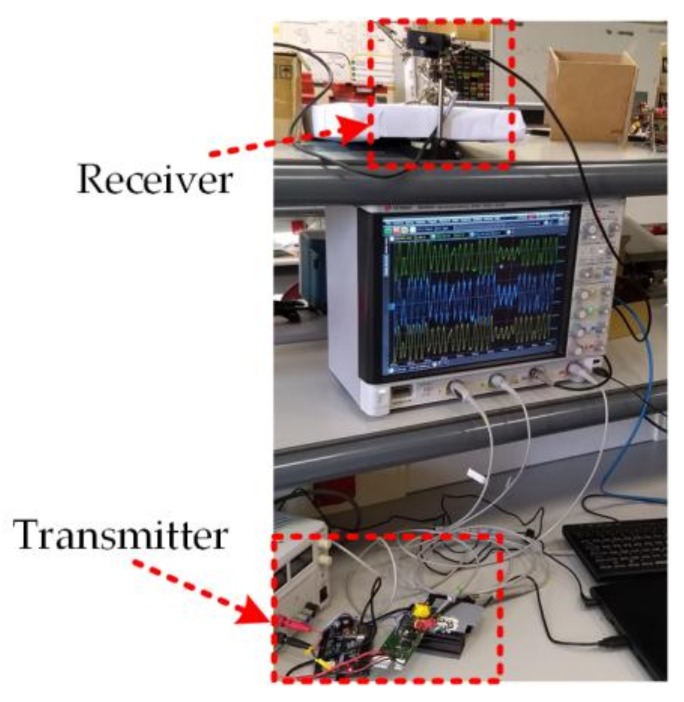
VLC setup transmitting the test sequence (the link distance is around 1 m).

Finally, Figure 17 shows the VLC setup, which is made up of the aforementioned transmitter, the receiver, and the oscilloscope showing the most significant transmission waveforms. The distance between the transmitter and the receiver is around 1 m. To achieve the previous results, the electrical power delivered by the LED driver is close to 10 W, achieving power efficiencies between 85% and 90%.

## 5. Conclusions

The use of an SMC_dc-dc_ as the LED driver of VLC transmitters is the key to keeping the power efficiency of SSL and to increase the power of the communication signal in order to achieve a higher range. However, the conventional SMC_dc-dc_ used in SSL does not achieve the bandwidth that VLC demands. Therefore, designs that are more sophisticated are mandatory to fulfill the VLC driving requirements. The buck converter is one of the most promising candidates for acting as the LED driver of the VLC transmitter. Nevertheless, the conventional buck design suffers from high switching losses due to the high ratio between its switching frequency (i.e., *f_s_*) and the maximum frequency of the communication signal that is going to be reproduced (i.e., *f_o−max_*). In this paper, three topologies derived from the conventional buck converter were reviewed and proposed as LED drivers for VLC: using high-order output filters in the SMC_dc-dc_, using several input voltage sources to supply the SMC_dc-dc_, and using several phases in the SMC_dc-dc_ structure. Moreover, hybrid topologies combining the three approaches can be used to reduce the gap even more.

As an example, the design of a two-phase buck converter with a fourth-order output filter supplying five LEDs was presented in the experimental section. This prototype reproduces a 16-QAM scheme, achieving a bit rate equal to 500 kbps and a power efficiency around 90%. Moreover, it is important to note that higher bit rates can be achieved by combining the three strategies presented in this paper in a more complex way (i.e., higher order filters, multiple input voltages, and multi-phase implementations). For instance, a more sophisticated LED driver for VLC based on the three buck-derived topologies can be found in [30,31]. The VLC transmitter reproduces a 64-QAM OFDM with *f_o−max_* equal to 3 MHz and the maximum bit rate is around 17 Mbps. The SMC_dc-dc_ topology is a floating two-phase buck converter with a fourth-order output filter. Note that a floating buck converter can be seen as a particular case of a two-input buck converter that considers (*v*_*in*−1_-*v*_*in*−2_) and *v*_*in*−2_ as inputs instead of *v*_*in*−1_ and *v*_*in*−2_ separately.

Finally, this paper has not only presented a solution to design an efficient LED driver for VLC, but also the authors’ views on the role that power electronics will play in VLC.

## Figures and Tables

**Table 1:** Amplitudes, phases, and bit codes of the reproduced 16-Quadrature Amplitude modulation (16-QAM).

Symbol	Normalized Amplitude	Phase (◦)	Bit Code	Symbol	Normalized Amplitude	Phase (◦)	Bit Code
*S*_1_	0.74	18.5	0000	*S*_9_	0.74	198.5	1000
*S*_2_	0.33	45	0001	*S*_10_	0.33	225	1001
*S*_3_	1	45	0010	*S*_11_	1	225	1010
*S*_4_	0.74	71.5	0011	*S*_12_	0.74	251.5	1011
*S*_5_	0.74	108.5	0100	*S*_13_	0.74	288.5	1100
*S*_6_	0.33	135	0101	*S*_14_	0.33	315	1101
*S*_7_	1	135	0110	*S*_15_	1	315	1110
*S*_8_	0.74	161.5	0111	*S*_16_	0.74	341.5	1111

**Table 2:** Reactive elements of the implemented fourth-order Legendre–Papoulis filter.

*L_1-a_* and *L_1-b_*	*C*_2_	*L*_3_	*C*_4_
3.4 μH	70 nF	1.5 μH	27 nF
